# Biophysical and structural characterization of a zinc-responsive repressor of the MarR superfamily

**DOI:** 10.1371/journal.pone.0210123

**Published:** 2019-02-12

**Authors:** Paloma Fernández Varela, Christophe Velours, Magali Aumont-Niçaise, Blandine Pineau, Pierre Legrand, Isabelle Poquet

**Affiliations:** 1 Synchrotron SOLEIL, L’Orme des Merisiers, Gif-sur-Yvette, France; 2 Laboratoire d’Enzymologie et Biochimie Structurales, CNRS Gif-sur-Yvette, France; 3 IMAGIF, CNRS Gif-sur-Yvette, France; 4 Institut de Biochimie et Biophysique Moléculaire et Cellulaire, Université Paris-Sud, Orsay, France; 5 Micalis Institute, INRA, AgroParisTech, Université Paris-Saclay, Jouy-en-Josas, France; Russian Academy of Medical Sciences, RUSSIAN FEDERATION

## Abstract

The uptake of zinc, which is vital in trace amounts, is tightly controlled in bacteria. For this control, bacteria of the *Streptococcaceae* group use a Zn(II)-binding repressor named ZitR in lactococci and AdcR in streptococci, while other bacteria use a Zur protein of the Ferric uptake regulator (Fur) superfamily. ZitR and AdcR proteins, characterized by a winged helix-turn-helix DNA-binding domain, belong to the multiple antibiotic resistance (MarR) superfamily, where they form a specific group of metallo-regulators. Here, one such Zn(II)-responsive repressor, ZitR of *Lactococcus lactis* subspecies *cremoris* strain MG1363, is characterized. Size Exclusion Chromatography-coupled to Multi Angle Light Scattering, Circular Dichroism and Isothermal Titration Calorimetry show that purified ZitR is a stable dimer complexed to Zn(II), which is able to bind its two palindromic operator sites on DNA fragments. The crystal structure of ZitR holo-form (Zn(II)_4_-ZitR_2_), has been determined at 2.8 Å resolution. ZitR is the fourth member of the MarR metallo-regulator subgroup whose structure has been determined. The folding of ZitR/AdcR metallo-proteins is highly conserved between both subspecies (*cremoris* or *lactis*) in the *Lactococcus lactis* species and between species (*Lactococcus lactis* and *Streptococcus pneumoniae or pyogenes*) in the *Streptococcaceae* group. It is also similar to the folding of other MarR members, especially in the DNA-binding domain. Our study contributes to better understand the biochemical and structural properties of metallo-regulators in the MarR superfamily.

## Introduction

Zinc is a widespread transition metal and, although Zn(II) cation is toxic at high concentrations, it is absolutely essential for life [1]. Zn(II) is necessary for many cellular functions including DNA replication, transcription, and translation. At a molecular level, it performs an essential structural role in protein or protein domain folding (e.g. zinc-finger proteins…), and it is also important for many enzymatic activities, as more than 300 enzymes bind Zn(II) [2].

To maintain cytoplasmic Zn(II) homeostasis, bacterial cells have to tightly control its transport. Zn(II) ABC uptake systems, which are of high affinity, allow acquisition of Zn(II) at very low concentrations, when it is present as a trace element in the environment [3, 4]. The expression of the operons encoding these ABC systems is under the control of efficient Zn(II)-responsive repressors, hereafter designated as ZnRR. When Zn(II) is abundant, holo-ZnRR are active repressors avoiding unnecessary uptake. On the contrary, when Zn(II) is limiting, apo-ZnRR are inactive, allowing efficient Zn(II) uptake to maintain intra-cellular homeostasis [3, 4]. ZnRR have been identified to date in only two protein superfamilies, Ferric uptake regulator (Fur) and Multiple antibiotic resistance (MarR). Fur superfamily exclusively consists of metallo-regulators specifically involved in the control of metal homeostasis and of very few related functions [5]. The wide majority of Fur members control the homeostasis of the metallic cation they bind by repressing its uptake (Fur, Mur, Nur and Zur proteins are named according to Fe(III), Mn(II), Ni(II) or Zn(II) cations respectively), while some other ones repress the uptake of iron-containing heme, and finally PerR control oxidative stress resistance [3, 4]. In marked contrast to Fur members, MarR superfamily members regulate much more numerous and diverse physiological functions, including for example the transport of antibiotics [6, 7]. In addition, even though both Fur and MarR proteins are α-helix proteins that exist as homo-dimers, they differ by a completely different folding. In Fur proteins, the winged-helix DNA binding domain is at the N-terminus while the regulatory metal binding and dimerization regions are located at the C-terminus [8]. In marked contrast, MarR members adopt a triangular shape [9]: both the N- and C-terminal α-helices form the dimerization region, and the central globular DNA-binding domain is folded as a winged helix-turn-helix (wHTH) domain [10]. In the long-known MarR superfamily, only a few recently identified members have been shown to be metallo-regulators involved in the control of metal homeostasis, and more precisely, to be ZnRR proteins controlling Zn(II) uptake [3, 4].

ZnRR of the MarR superfamily have only been found in *Streptococcaceae*, a bacterial group including *Streptococcus* and *Lactococcus* genera, and have respectively been named AdcR [11–14] and ZitR [15, 16]. AdcR was the first MarR regulator shown to control genes encoding an ABC system for Zn(II) uptake [11, 12, 17]. In the pathogenic species *Streptococcus pneumoniae* and in the dairy species *Lactococcus lactis*, *adcR* and *zitR* regulatory genes are co-organized as operons with their *adc-zit* target genes, which encode ABC uptake systems specific for Zn(II) [11, 12, 15, 17]. In *L*. *lactis* subspecies *cremoris* strain MG1363, we have showed that ZitR repressor controls an emergency response to Zn(II) starvation, allowing Zn(II) uptake under extreme conditions, while impeding it in a wide concentration range, from repletion to toxicity [16]. At the molecular level, both AdcR protein of *S*. *pneumoniae* [14, 18] and ZitR protein of *L*. *lactis* subspecies *cremoris* [15, 16] have been shown to bind one or two conserved palindromic **TTAAC**YR**GTTAA** operator sites [19] in the promoter sequence of *adc*/*zit* operon and other genes. Furthermore, ZitR/AdcR proteins assemble as homo-dimers, with a stoichiometry of two cations/protomer [14, 16]. The affinity of AdcR for Zn(II) ranges from pM to nM [18]. During the course of this study, the structures of three ZnRR of the MarR superfamily have been determined. *S*. *pneumoniae* AdcR protein (hereafter designated as AdcR_Spne_) has first been shown to be a holo-dimer with two Zn(II) ions bound per protomer [13]. *S*. *pyogenes* AdcR protein (hereafter designated as AdcR_GAS_) has then been confirmed to be folded in a highly similar way [20]. Recently, ZitR protein of *L*. *lactis* subsp. *lactis* strain IL1403 (hereafter designated as ZitR_IL_) has been described as a holo-dimer complexed or not to DNA [21].

This work was aimed to more deeply characterize, from a biochemical and structural point of view, ZitR protein of *L*. *lactis* subspecies *cremoris* strain MG1363 (hereafter designated as ZitR_MG_), which we have previously studied both *in vitro* and *in vivo* [16]. After protein production and purification in the presence of Zn(II) as previously described [16], we measured the protein stability in solution by Circular Dichroism (CD), and determined its oligomeric state by Size Exclusion Chromatography-coupled to Multi Angle Light Scattering (SEC-MALS). We also confirmed ZitR_MG_ ability to bind its *zit* DNA operator site using Isothermal Titration Calorimetry (ITC). X-ray crystallography was finally used to determine the protein 3D structure. Our study allows increasing the knowledge about biophysical and structural properties of ZnRR proteins belonging to the MarR superfamily.

## Materials and methods

### ZitR purification and characterization

An untagged, recombinant (S2A, A4R, D8E) form of ZitR protein (145 amino acids, full length) from *L*. *lactis* subsp. *cremoris* strain MG1363 (UniProtKB/Swiss-Prot A2RNS2) was produced and purified as previously described [16]. Recombinant ZitR_MG_ protein was first over-produced in *Escherichia coli* strain (BL21(DE3) (pVE8073) at 25 °C [16]. It was then purified in the presence of ZnCl_2_ in two steps by anion exchange chromatography followed by heparin-affinity chromatography [16]. SDS-PAGE analysis revealed the presence of a few high molar mass contaminating *E*. *coli* proteins (data not shown), which could be eliminated by gel filtration chromatography in 20 mM Tris-HCl (pH 7.0), 200 mM NaCl and 100 μM ZnSO_4_. At the end of this purification process, we observed a major dimeric form, and a minor tetrameric form (data not shown). Except for preliminary experiments (see below), only the major dimeric form has been studied and is described here. http://dx.doi.org/10.17504/protocols.io.vgye3xw.

#### Matrix-Assisted Laser Desorption/Ionization—Time of flight mass spectrometry (MALDI-TOF)

For protein identification after SDS-PAGE, bands of interest were cut, submitted to trypsynolysis and analyzed using MALDI-TOF/TOF 5800 (AB Sciex) (SICaPS platform, IMAGIF). Comparison to NCBI database sequences using Mascot confirmed that the purified protein was ZitR protein from lactococcal strain MG1363 (UniProtKB/Swiss-Prot A2RNS2) (data not shown). http://dx.doi.org/10.17504/protocols.io.vgze3x6.

#### Fluorescence-based Thermal Shift Assay (TSA)

Thermal stability of fully purified ZitR_MG_ protein was calculated by induced thermal denaturation using a Q-PCR ABI Prism 7900HT (CTPF platform, IMAGIF). We used Sypro Orange dye, which non-specifically binds to hydrophobic surfaces and can be measured at 488 nm. Purified ZitR_MG_ protein (2.5 μg) was analyzed under different conditions of buffer (Tris, phosphate, HEPES and MES), pH values (ranging from 9.2 to 4), and salt (NaCl up to 350 mM) or glycerol concentrations (up to 10%) (data not shown). http://dx.doi.org/10.17504/protocols.io.vg2e3ye.

#### Size Exclusion Chromatography coupled to Multi Angle Light Scattering (SECMALS)

To determine the oligomeric state of ZitR_MG_, 50 μl of fully purified protein at approximately 1–4 mg.ml^-1^ were loaded on a KW-803 column (Shodex) equilibrated at 0.5 ml.min^-1^ flow rate (Shimadzu HPLC system) and supplemented with either 1 μM ZnSO_4_ or 1 mM EDTA (S1 Fig). Detection was performed using a MiniDAWN TREOS multi angle light scattering detector and an Optilab T-rEX differential refractometer (Wyatt Technology) (biophysics platform, LEBS/IMAGIF). Molar mass and hydrodynamic radius were calculated with the Astra 6.1.1.17 software using a differential index of refraction (dn/dc) value of 0.183 ml.g^-1^. http://dx.doi.org/10.17504/protocols.io.vg3e3yn.

#### Circular dichroism

Synchrotron radiation circular dichroism (SRCD) experiments were carried out at 15 °C on the DISCO beamline (SOLEIL synchrotron). D-10-camphorsulfonic acid was used to calibrate the SRCD signal using the CDtool software [22]. Spectra were obtained using calcium fluoride circular cells (Hellma) of 50 μm path length. They were loaded with ZitR_MG_ protein (8 μg) in 20 mM Tris-HCl (pH 7.0), 50 mM NaCl and 100 μM ZnSO_4_ buffer. Acquisitions at 1 nm step per second between 170 to 305 nm were recorded in triplicates. Averaged spectra were corrected with respect to the baseline by buffer subtraction and set to zero in the 300–305 nm region (S2 Fig).

Standard circular dichroism (CD) measurements were carried out at 20 °C on a JASCO J-810 spectropolarimeter. Temperature was controlled by a Peltier (Jasco PFD423S/L) (biophysics platform, LEBS/IMAGIF). Spectra from 185 to 260 nm were obtained using a 100 μm path length suprasil quartz cell (Hellma) containing ZitR_MG_ protein (400 μg) in 20 mM Tris-HCl (pH 7.0), 50 mM NaCl and 100 μM ZnSO_4_ buffer. All data processing was performed using CDtool software and secondary structure prediction of ZitR_MG_ protein was carried out on Dichroweb server (http://dichroweb.cryst.bbk.ac.uk) using all available algorithms (CONTINLL, SELCON3, CDSSTR and K2D3) and all sets of proteins (database 1–7, SP175, SMP180) [23, 24] http://dx.doi.org/10.17504/protocols.io.vg4e3yw.

### Holo-ZitR_MG_ binding to dsDNA fragments

The interaction of fully purified ZitR_MG_ protein with dsDNA of different sizes (19- and 20-mers), each containing one DNA-binding domain [16, 19], was explored. Complementary oligonucleotides (from SIGMA, Eurogentec and Invitrogen) containing an imperfect **TTAAC**YR**GTTAA** palindrome overlapping either the -35 or the -10 box of the ZitR-controlled promoter region (see S1 Table for the sequences of forward oligonucleotides) were purified by SDS-PAGE or desalting. 5’ overhang nucleotides (adenine in the forward oligonucleotide and thymine on the complementary one) were added to the sequence in order to make the dsDNA stickier, which might help in crystallization. Annealing of complementary forward and reverse oligonucleotides were carried out by incubation at 95 °C during 5 min in a 20 mM Tris-HCl (pH 8) and 150 mM NaCl buffer, followed by incubation on ice to slowly decrease the temperature.

Isothermal Titration Calorimetry (ITC) was performed on a Microcal ITC200 (GE Healthcare) (calorimetry platform, IBBMC/IMAGIF). Purified ZitR_MG_ protein was dialyzed against a 20 mM Tris-HCl (pH 7.0), 150 mM NaCl and 100 μM ZnSO_4_ buffer. Duplicate titration of approximately 20 μM ZitR_MG_ protein, while stirring at 1000 rpm, was carried out by 20 injections of 2 μl of each dsDNA at 270 μM in the same buffer as the protein. The heat generated by DNA dilution was determined from the peaks measured after full saturation of the protein. Experimental data were fitted to the theoretical titration curves using the Origin software (OriginLab, Northampton, MA) according to the relationship between the heats generated by each injection. The following values were calculated: ΔH_cal_, enthalpy change in kcal.mol^-1^; K_a_, association-binding constant in M^-1^; n, number of binding sites. The binding constant of each interaction is expressed as 1/K_a_ = K_d_ (in mol.L^-1^) (S3 Fig). http://dx.doi.org/10.17504/protocols.io.vg5e3y6.

### Holo-ZitR_MG_ structure determination

Holo-ZitR_MG_ protein was subjected to crystallization assays using spare matrix screens in a robotic system (Nano-Robot Cartesian) (crystallography platform, LEBS/IMAGIF). In preliminary experiments, crystals were first obtained using ZitR_MG_ tetrameric form by vapor diffusion in 0.2 M ammonium acetate, 0.1 M sodium citrate tribasic dihydrate (pH 5.6) and 30% polyethyleneglycol 4,000. They diffracted to a resolution of less than 6 Å. These crystals belong to *P*4_1_ space group with cell parameters of *a* = *b* = 129 Å; *c* = 88 Å; α = β = γ = 90°. Crystallization conditions were then optimized using the dimeric form. X-ray data were collected at the zinc peak absorption edge up to 3.7 Å resolution on the PROXIMA-1 beamline at SOLEIL synchrotron. After data processing by XDS [25], molecular replacement with coordinates of different MarR proteins was unsuccessful. We therefore used Zn(II) anomalous signal to solve the structure by SAD method using PHASER [26]. Eight molecules were found in the asymmetric unit, with a solvent content of 60%, and two zinc atoms were found per protomer. Phases were improved and extended by NCS averaging and solvent flattening using PARROT [27]. Refinement was done using BUSTER-TNT [28]. A low-resolution model could be built using *Bacillus subtilis* OhrR structure [29]. We could identify the extended wHTH DNA-binding domain from a poly-Ala chain, but most of the lateral chains were not visible. New data collection recorded on a Pilatus 6M detector on the same beamline went up to 2.8 Å resolution. An anisotropic correction factor was applied by STARANISO server (http://staraniso.globalphasing.org/) to sharpen the data in the weaker-diffracting direction. Structure was determined by molecular replacement using *S*. *pneumoniae* AdcR (3TGN) as a model [13]. The high values of average B-factors of the final structure can be explained by the crystal anisotropy. All structure figures have been created using The PyMOL Molecular Graphics System, Version 1.8 Schrödinger, LLC http://dx.doi.org/10.17504/protocols.io.vg6e3ze.

## Results and discussion

### ZitR protein of *L*. *lactis* subsp *cremoris* strain MG1363 is a representative member of ZnRR in the MarR superfamily

**Fig 1:**
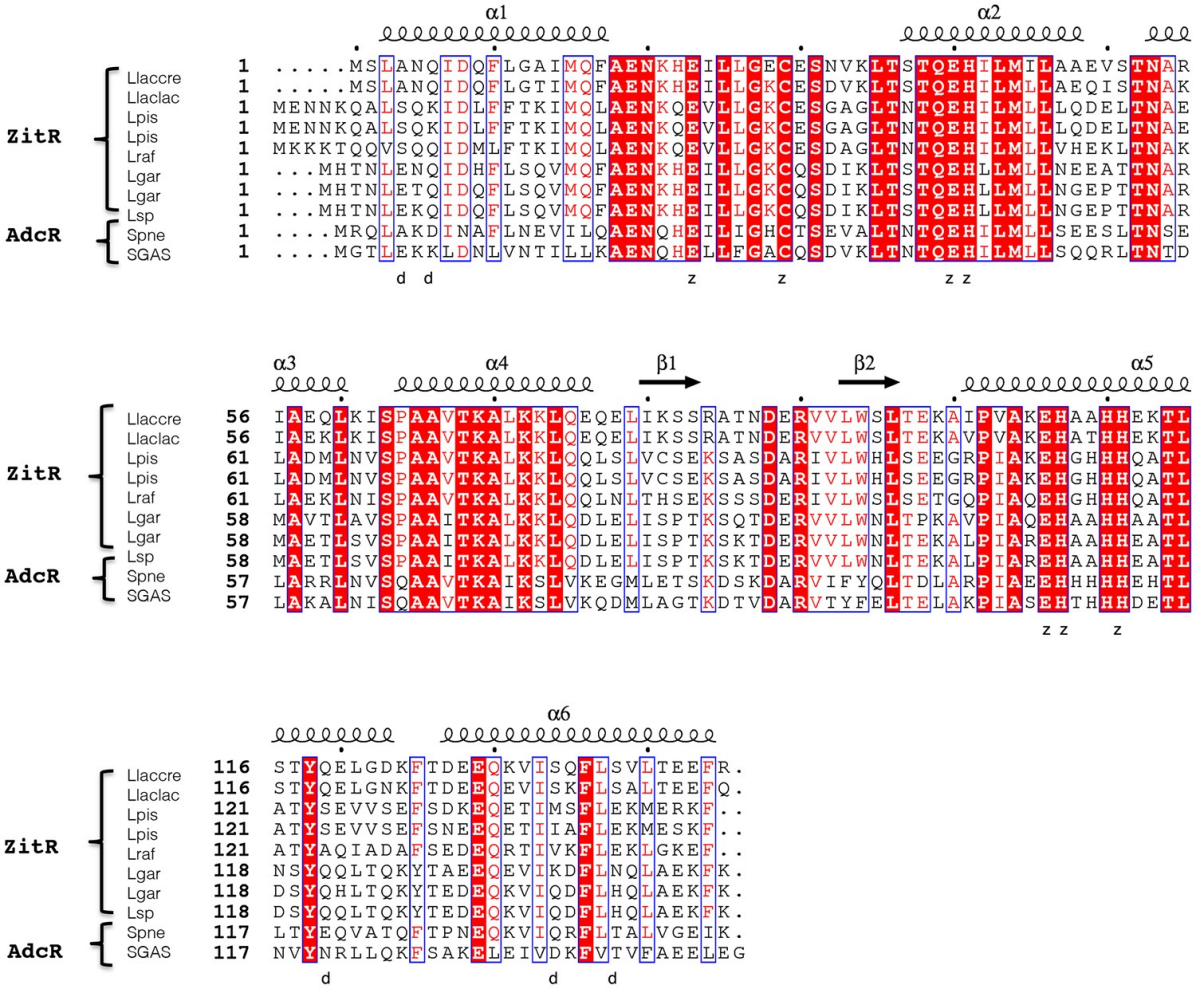
Sequence conservation between ZitR/AdcR proteins. Multi-alignment of ZitR/AdcR protein sequences was performed using Clustal Omega server (https://www.ebi.ac.uk/Tools/msa/clustalo/) and ESPript server (http://espript.ibcp.fr/ESPript/ESPript/) [30]. We noticed that several lactococcal proteins are erroneously annotated as ‘AdcR’ in gene/protein databases, as they share a higher homology level with ZitR proteins than to the prototypal AdcR protein from *S*. *pneumoniae* [13, 18, 19]. We therefore renamed them as ZitR proteins. The following sequences are shown: i) ZitR from *L*. *lactis* subsp. *cremoris* (Llaccre) strain MG1363 (gb |A2RNS2.1|), our lactococcal protein model [15, 16]; ii) ZitR from *L*. *lactis* subsp. *lactis* (Llaclac) strain IL1403 (gb |NP_268273.1|, gb |AAK06214.1|, gb |Q9CDU5|, PDB: 5YHX [21]), which is identical to ZitR proteins from *L*. *lactis* subsp. *lactis* strain NCDO 2118 (gb |AII13676.1|) and subsp. *lactis* bv. *diacetylactis* (gb |KZK11188.1|), not shown; iii) ZitR (‘AdcR’) from *L*. *piscium* (Lpis) strains MKFS47 (gb |CEN27435.1|) and iv) CNCM I-4031 (gb |SCA91076.1|); v) ZitR (‘AdcR’) from *L*. *raffinolactis* (Lraf) strain 4877 (gb |CCK19333.1|); vi) ZitR (‘AdcR’) from *L*. *garvieae* (Lgar) strains DCC43 (gb |EKF52529.1|) and vii) 122061 (gb |BAV02535.1|), which is identical to the protein from strain M14 (gb |CEF51164.1|), not shown; viii) ZitR (‘AdcR’) from *Lactococcus* sp. (Lsp) strain DD01 (gb |KXT61547.1|); ix) the prototypal AdcR protein [13, 18] from *S*. *pneumoniae* (Spne) strain D39 (gb |Q04I02.1|, PDB: 3TGN [13]); and finally x) AdcR protein from *S*. *pyogenes* strain MGAS315 (gb |AAM78676.1|), or serotype M3 (PDB: 5JLU [20]). Secondary structure elements of ZitR_MG_ protein are displayed above its sequence: α-helices as medium squiggles and β-strands as arrows. ZitR_MG_ residues shown here (see below) to belong to the dimerization interface or to the metal binding pocket (Fig 2) are respectively indicated by ‘d’ and ‘z’ characters below the sequences. Amino acids that appear in white characters in a red background are identical in all aligned proteins, while those in red characters and in blue frames are conserved in the majority of proteins.

In the whole *Streptococaceae* group, ZnRR of the MarR superfamily are known to be conserved and ZitR/AdcR proteins are 48% identical (Fig 1). In the *Streptococcus* genus, AdcR proteins are slightly more conserved and display around 50% identity among species and even more within species [16]. The same holds true for lactococcal ZitR proteins, with more than 55% identity between species in the *Lactococcus* genus (e.g. more than 65% identity between *L*. *lactis* and *L*. *garvieae* proteins) and up to 89% identity between *cremoris* and *lactis* subspecies. ZitR_MG_ [15, 16] appears to be a representative member of ZitR proteins among lactococcal species (Fig 1) and was chosen to be studied here. It is the fourth ZnRR of the MarR superfamily [13, 20] and the second lactococcal one [21] whose structure is determined.

### ZitR_MG_ protein is a stable Zn(II)-bound dimer able to bind its DNA operator site

We produced a recombinant, untagged form of ZitR_MG_ protein (145 amino acids, 16.3 kDa) in *E*. *coli*, purified it in the presence of Zn(II) as previously described [16], and analyzed its secondary structure. CD spectra demonstrated that purified ZitR_MG_ contained a majority of helices (73.7%) and a few strands (11%). This is in agreement with ZitR_MG_ protein being predicted to contain six α-helices and two antiparallel β-hairpins per protomer, as all other MarR superfamily members [7, 9]. We also determined the oligomeric state of purified ZitR_MG_ by SEC-MALS. Its molar mass was found to be 32.1 ± 0.3 kDa (S1 Fig) as expected for a dimer. The protein hydrodynamic radius was found to be 2.3 ± 0.1 nm (data not shown) denoting a globular protein. EDTA, a chelator of cationic ions and in particular of Zn(II), could be added to ZitR_MG_ without significantly modifying its molar mass (S1 Fig), therefore confirming that Zn(II) is not required for ZitR_MG_ dimerization [16]. Overall, purified ZitR_MG_ protein appeared to be both a typical MarR superfamily member, able to form stable dimers mainly folded as α-helices [6, 9], and a typical ZnRR able to bind Zn(II).

We then verified that ZitR_MG_ protein was in its holo-form as expected [16]. Synchrotron Radiation CD (SRCD) spectroscopy and Fluorescence-based Thermal Shift Assay (TSA) were first used. Both revealed that purified ZitR_MG_ protein had a high melting temperature (Tm) of around 75 °C, indicative of a stable protein (S2 Fig, TSA data not shown). When EDTA was added in excess to the protein, a much lower Tm of around 45 °C was measured by TSA (data not shown). These results confirm that purified ZitR_MG_ protein is complexed to divalent (metallic) cations [16] whose binding is required for protein stability. ZitR_MG_ protein has been found to be very unstable in the presence of EDTA, so that its apo-form could not be further characterized.

We also verified whether purified ZitR_MG_ protein could bind DNA as expected [16]. Purified ZitR_MG_ has previously been shown to be able to bind two imperfect **TTAAC**YR**GTTAA** palindromic sequences (with the two 5 bp-inverted repeats in bold) that are present in *zit* promoter (P*zit*) and overlap its -35 and -10 boxes [16]. Two double-stranded (ds) small DNA fragments, each containing one palindromic sequence, were designed to perform binding assays using purified ZitR_MG_ (S1 Table). ITC experiments confirmed, as expected, that ZitR_MG_ protein was able to bind its two palindromic sequences (S3 Fig) [16]. They also revealed positive enthalpy changes (ΔH), indicating that ZitR_MG_ interaction with dsDNA fragments is an endothermic and entropically driven process that implies a hydrophobic component in the binding.

### Crystal structure of holo-ZitR_MG_ protein

The structure of ZitR_MG_ holo-form was first determined at low resolution by single anomalous wavelength dispersion (SAD) and then, after crystal improvement by molecular replacement, at 2.8 Å resolution. The asymmetric unit (ASU) contained 8 ZitR_MG_ molecules, and 16 Zn(II) atoms. ZitR_MG_ molecules formed four dimers (AB, CD, EF and GH), all binding four Zn(II) atoms in total (two per protomer). Crystallographic data and refinement statistics are summarized in Table 1. Three of the dimers were almost identical, with all 2290 atoms root-mean-square-deviation (RMSD) values ranging from 0.34 Å (AB/CD) to 0.50 Å (AB/GH). Dimer EF was less defined (with high all 2290 atoms RMSD values, at least 1.37 Å (AB/EF) and up to 1.44 Å (EF/GH)). AB dimer was chosen as a ZitR_MG_ dimer prototype and is described in the following. In AB, both protomers have almost the same conformation and superimpose to each other with all 1144 atoms-RMSD of 0.34 Å. Each protomer is arranged from the N- to the C-terminus as follows: α1 (residues 3–17), α2 (residues 37–48), α3 (residues 53–60), α4 (residues 64–77), β1 (residues 80–83), β2 (residues 93–96), α5 (residues 100–124) and α6 (residues 127–144) (Fig 1), in good agreement with our above-mentioned CD results, and as expected for a typical MarR superfamily member [7, 9].

**Table 1:** Crystallographic data and statistics.

***Data Collection***	
Space group	P 41
*a*, *b*, *c* (Å)	128.52, 128.52, 88.14
α,β,γ (°)	90, 90, 90
Wavelength (Å)	1.28189
Resolution (Å)	48.15–2.8 (2.975–2.872)^h^
No. of observed reflections	43447 (1029)^h^
No. of unique reflections	21630 (515)^h^
Multiplicity	2.0 (2.0)^h^
R_*merge*_ (%)^a^	6.722 (51.34)^h^
R_meas_ (%)^b^	9.506
Completeness (%)	65.60 (13.34)^h^
I/σ^c^	16.62 (1.62)^h^
CC_1/2_^d^	0.994 (0.392)^h^
CC*^e^	0.998 (0.751)^h^
***Refinement***	
R_*work*_ (%)^f^	23.65
R_*free*_ (%)^g^	25.14
Number of non-hydrogen atoms	9168
in macromolecules	9152
in ligands	16
Protein residues	1152
RMS bond deviation (Å)	0.012
RMS angle deviation (°)	1.57
Ramachandran favored (%)	97.98
Ramachandran allowed (%)	2.02
Ramachandran outliers (%)	0
Clashcore	3.08
Average B-factor	162.20
in macromolecules	162.20
in ligands	160.60

^a^Rmerge = ∑_hkl_∑_j_|I_hkl,j_-<I_hkl_>|∑_hkl_∑_i_I_hkl,j_

^b^Rmeas = ∑_hkl_√n/n-1 ∑^ν^_j = 1_|I_hkl_-<I_hkl_>|∑_hkl_∑_i_|I_hkl,j_

^c^I/ σ = signal-to-noise ratio

^d^CC_1/2_ = Pearson correlation coefficient

^e^CC* = √2CC_1/2_/1+CC_1/2_

^f^Rwork = ∑_hkl_∥Fobs(hkl)|-k|Fcalc(hkl)∥/ ∑_hkl_|Fobs(hkl)

^g^Rfree = ∑_hklCT_∥Fobs(hkl)|-k|Fcalc(hkl)∥ ∑_hklCT_|Fobs(hkl)

^h^Values in parenthesis correspond to the last resolution shell

**Fig 2:**
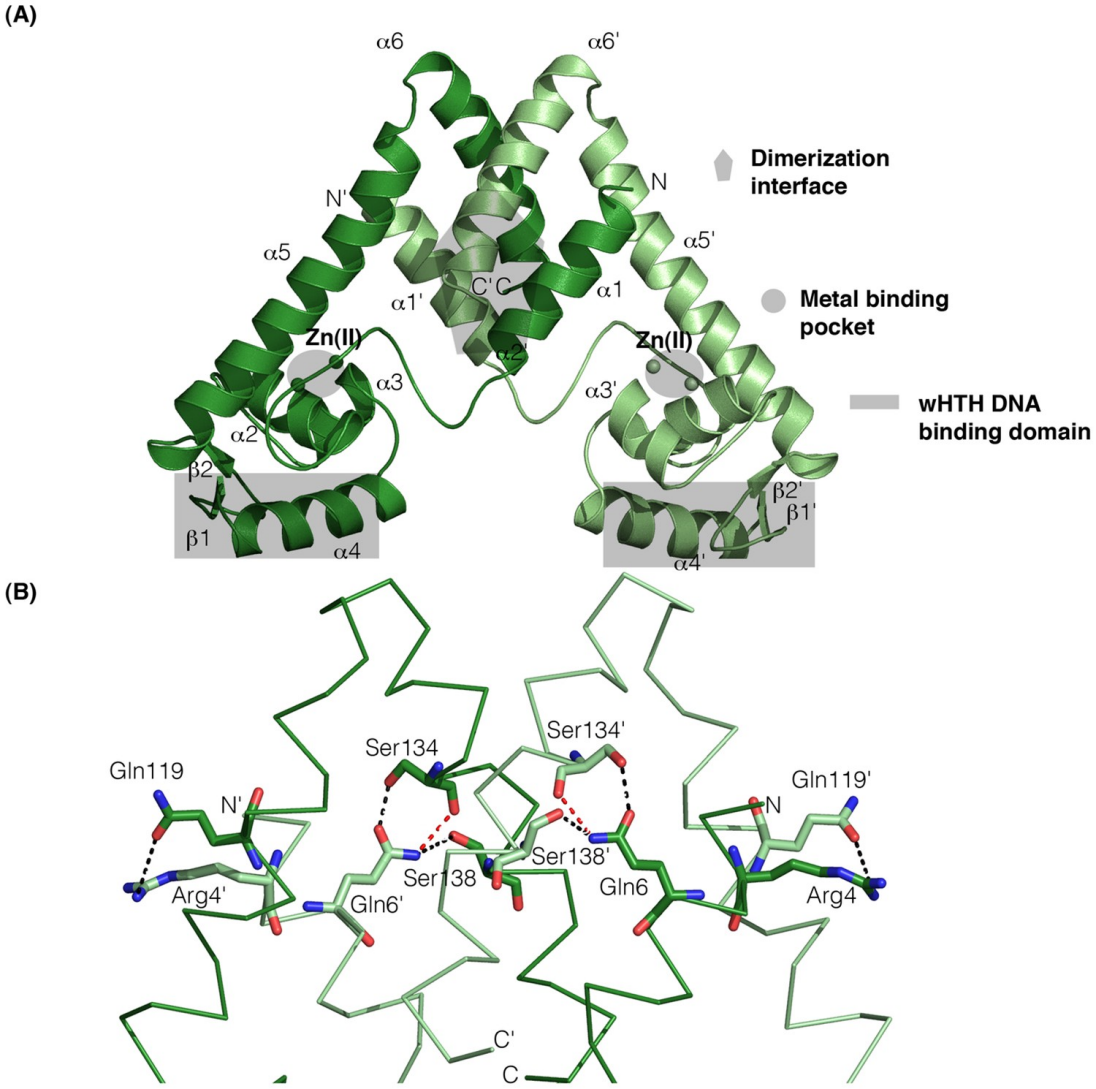
Structure of holo-ZitR_MG_ dimer. (A) Overall structure. Protomers A and B of ZitR_MG_ dimer, represented in ribbon, are colored in dark and light green respectively, and their N- and C-terminus are indicated. Zn(II) atoms are shown as small green spheres. Helices and strands are labelled. The dimerization interface, each wHTH DNA-binding domain and each metal-binding pocket are indicated by different forms colored in grey, respectively a polygon, a rectangle and a circle. (B) Close view of dimer interface. Protomers A and B of ZitR_MG_ dimer are shown as in Fig 2A. Residues involved in dimerization are labelled and represented in ball-and-sticks, with N atoms colored in blue and O atoms in red. Hydrogen bonds are shown as dashed lines, either in black in most cases or in red when they involve main chain atoms.

ZitR_MG_ protein is folded as a triangular-shaped homo-dimer with a two-fold pseudo-symmetric axis (Fig 2A), a typical topology for a MarR superfamily member [7, 10]. It best matches ZitR_IL_ holo-dimer, but it also well aligns to the DNA-bound form of ZitR_IL_ [21], the holo-form of streptococcal AdcR proteins [13, 20], and finally non-ZnRR members of the MarR superfamily (Table 2) (S4A and S4B Fig). Each monomer of ZitR_MG_ protein contains three well-defined regions (in grey in Fig 2A): a dimerization interface, a wHTH DNA-binding domain and a metal-binding pocket, which are described in the following sections.

**Table 2:** Structure comparison between ZitR_MG_ protein and other MarR superfamily members.

Protein or Protein/ DNA complex (PDB)	Species	Protein Identity to ZitR_MG_ (%)	r.m.s.d over all atoms protomer	r.m.s.d over all atoms dimer	Reference
ZitR_MG_ (this study, 6FI9)	*L*. *lactis* subsp. *cremoris* (strain MG1363)	100	0.34	***Not Applicable***	This study
ZitR_IL_ (5YHX)	*L*. *lactis* subsp. *lactis* (strain IL1403)	89	1.76	**1.18**^a^	[21]
ZitR_IL_/16-mer DNA (5YI2)			2.41	**2.30**^a^	
AdcR_Spne_ (3TGN)	*S*. *pneumoniae*	47	**1.65**^a^	2.76	[13]
AdcR_GAS_ (5JLS)	*S*. *pyogenes*	44	**3.35**^a^	6.01	[20]
AdcR_GAS_ (C-terminally His tagged, 5JLU)			**4.07**^a^	6.42	
OhrR/29-mer DNA (1Z9C)	*B*. *subtilis*	23	**5.87**^a^	6.51^b^	[29]
OhrR (1Z91)			**17.03**^a^	17.16	
RovA /21-mer DNA (4AIJ)	*Y*. *pseudotuberculosis*	24	**4.83**^a^	6.09	[31]
RovA (4AIH)			**5.28**^a^	7.19^b^	
SlyA/23-mer DNA (3Q5F)	*S*. *enterica*	22	**4.91**^a^	6.06	[32]
SlyA (3QPT)			**5.37**^a^	8.17	

^a^For each comparison the best RMSD value is in bold.

^b^When several dimers are present in the asymmetric unit of a protein crystal (ohrA-bound OhrR (1Z9C) and DNA-free RovA (4AIH)) only the AB dimer is considered in this Table.

#### Protomers interact via hydrogen bonds in the dimerization interface

The dimerization interface involves the N- and C-terminus of ZitR_MG_ protein, as in ZitR_IL_ [21] and AdcR proteins [13, 20]. In ZitR_MG_ dimerization interface, the N-terminal α1 helix (protomer A), which is stacking between α5'-α6' helices, interacts with α1’, its counterpart in protomer B, and the same holds true for the C-terminal α6 and α6' helices (Figs 1 and 2A). These interactions are mainly through four hydrogen bonds (Fig 2B). Three of them, involving the following atom pairs: Nε2-Gln6 (α1) and main chain O-Ser134’, Nε2-Gln6 and Oγ-Ser138’ (α6'), and finally Oε1-Gln6 (α1) and Oγ-Ser134’ (α6'), are conserved in ZitR_IL_ protein, while the fourth, NH1-Arg4 (α1) and Oε1-Gln119’ (α5'), is specific to the recombinant ZitR_MG_ protein (see Materials and methods section). AdcR proteins display three completely different hydrogen bonds. They involve: i) NZ-Lys7 (α1) and Oδ1-Asp135’ (α6'), Oε1-Glu20 (loop α1-α2) and N-Asn38’ (α2), and finally Oε2-Glu20 (loop α1-α2) and Oγ1-Thr39’ (α2), in the case of AdcR_GAS_ [20]; and ii) N-Arg2 (α1) and Oε1-Glu120’ (α5'), Nε-Arg2 (α1) and Oε1-Gln131’ (α6'), and finally Oε2-Asp7 (α1) and Nε2-Gln135 (α6'), in the case of AdcR_Spne_ [13]. In ZitR_MG_ dimer, the hydrogen bonds in the dimerization interface are probably involved in protein stability, as it is the case in the other ZitR/AdcR proteins of determined structure [13, 20, 21].

In the overall ZitR_MG_ structure, the dimerization interface and the DNA-binding domain are connected *via* the α2 and α5 helices (Fig 2A), as it is also the case in other MarR superfamily members [10].

#### The DNA-binding domain is structurally conserved between ZitR_MG_ and other MarR superfamily members

In ZitR_MG_ like in *E*. *coli* MarR [10], streptococcal AdcR proteins [13, 20] and ZitR_IL_ [21], the DNA-binding domain is folded as a wHTH domain made of a helix region (formed by α2, α3 and α4 helices) and a β strand region (β1 and β2 strands) (Fig 2A and S4A and S4B Fig). In all proteins of the MarR superfamily, these two regions are respectively involved in the binding of the DNA major and minor grooves [9]. The α4 helix and the β1 strand form an angle of approximately 30 ° in each ZitR_MG_ protomer, as it is the case in the other ZitR/AdcR proteins [13, 20, 21]. This conformation is widely conserved in MarR superfamily and probably allows DNA accommodation and binding [9].

In order to get insights about DNA-binding ability of ZitR_MG_, we compared its 3D fold to that of the three MarR members whose structure had been determined in both DNA-bound or DNA-free conformations at that time (and later also to that of ZitR_IL_ [21]): *Yersinia pseudotuberculosis* RovA [31], *Salmonella enterica* SlyA [32], and *B*. *subtilis* OhrR [29] (Table 2). It is worth noting that in these three proteins, DNA-binding activity is not induced by the binding of a ligand, in contrast to the situation in the best known superfamily member, *E*. *coli* MarR [9, 10]. Indeed, RovA and OhrR, which do not bind any ligand, acquire their DNA-binding activity upon a conformational change induced by a temperature [31] or oxygen shift respectively [29, 33], while in SlyA, the salicylate ligand and DNA compete for the same binding site [32]. Interestingly, ZitR_MG_ is shown here to better align to the DNA-bound form of any MarR protein than to its DNA-free counterpart (Table 2), suggesting that holo-ZitR_MG_ dimer could be folded in a DNA-binding prone conformation. Consistently, we previously showed by EMSA [16] that Zn(II) binding is required for the DNA-binding activity of purified ZitR_MG_ dimer, as also shown for AdcR proteins [14, 34]. In addition, in ZitR_IL_ protein, both DNA-bound and DNA-free holo-forms share a highly similar structure that is different from that of the apo-form [21], showing that only the holo-form is in a DNA-binding prone conformation. Finally, in ZnRR proteins of the MarR superfamily, activity is acquired upon the binding of the ligand, as it is the case for a distantly related, non ZnRR superfamily member, *E*. *coli* MarR. In this protein, ligand binding changes the orientation of the two DNA recognition regions and their distance in the wHTH domain [9].

**Fig 3:**
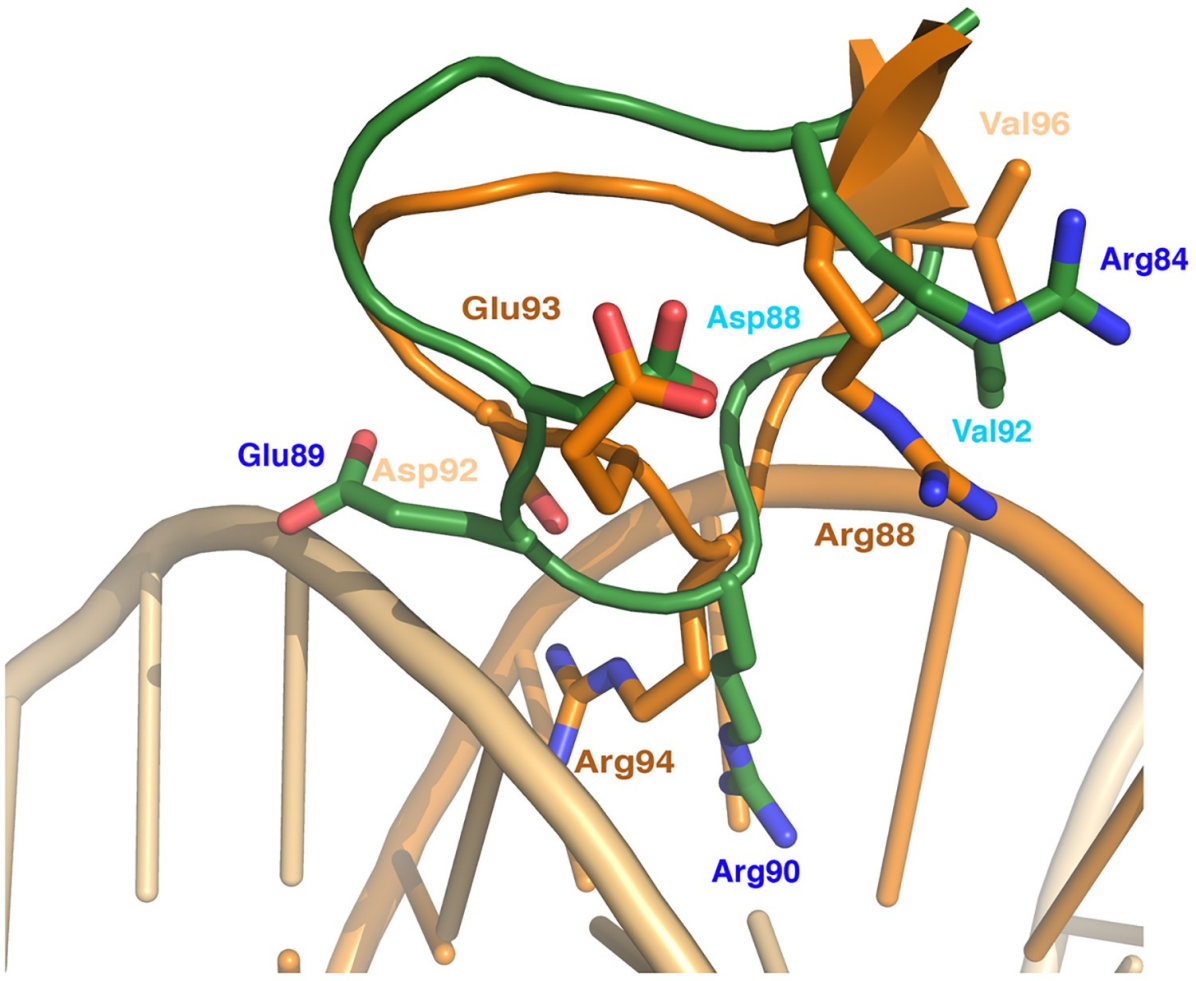
The wHTH DNA-binding domain of ZitR_MG_. The structure of holo-ZitR_MG_ protein (this study, 6FI9, protomer A is shown as a green ribbon) and that of OhrR-*ohrA* complex (1Z9C, OhrR protomer A is shown as an orange ribbon and *ohrA* DNA colored in light-orange) have been superimposed, and a zoom on the Arg94 of OhrR wHTH DNA-binding domain is shown. Residues aligned between ZitR_MG_ and OhrR are represented as ball-and-sticks with N colored in blue and O in red. Fully and moderately accessible residues are labelled in dark and light color respectively.

In ZitR_MG_ wHTH domain, we noticed that Arg90_ZitR_, a highly exposed residue, protrudes at the protein surface (Fig 3, S5A and S5B Fig), suggesting that it could interact with DNA. Noteworthy, Arg90_ZitR_ is widely conserved in all members of the MarR superfamily, including OhrR [29], RovA [31], SlyA [32], AdcR [13, 20] and ZitR_IL_ [21] (Fig 1 and S6 Fig), and this conserved Arg residue has been shown, in two superfamily members, MarR and MexR proteins, to be required for full DNA-binding activity *in vitro* [10, 35]. Furthermore, in the DNA-bound form of RovA [31], SlyA [32] and OhrR [29], this conserved Arg has been found to contact the DNA minor groove. This contact can neither be observed in DNA-bound ZitR_IL_, as DNA is too short, nor can it be excluded [21]. In OhrR [29], Asp92_OhrR_, which is also widely conserved in the whole MarR superfamily, notably in RovA [31], SlyA [32] and ZitR/AdcR proteins [13, 20, 21], has been shown to strengthen the interaction between Arg94_OhrR_ and DNA. In ZitR_MG_, the conserved Arg residue (Arg90_ZitR_) could therefore be located in the DNA minor groove with the help of the conserved Asp residue (Asp88_ZitR_). Besides, in OhrR, these two residues form a small cluster with three very close residues also contacting the DNA minor groove, Arg88_OhrR_, Glu93_OhrR_ and Val96_OhrR_ [29]. Interestingly enough, these three residues are specifically conserved in the sequences of ZitR_MG_ and ZitR_IL_ proteins (as Arg84_ZitR_, Glu89_ZitR_ and Val92_ZitR_ in ZitR_MG_), but not in that of other ZitR/AdcR proteins (Fig 1) or other MarR members (S6 Fig). In structure superimposition, they align between ZitR_MG_ and either OhrR (Fig 3) or ZitR_IL_ (S5 Fig), suggesting that the whole cluster around the Arg residue in the two crystallized ZitR proteins could define a wHTH region interacting with DNA minor groove. Finally, another ZitR_MG_ residue, Thr36_ZitR_ (loop α1-α2), both highly exposed at the protein surface and conserved in MarR superfamily members (S6 Fig), could also contact DNA by alignment to the conserved and DNA-contacting Thr residue in OhrR [29], RovA [31] and SlyA [32]. This Thr residue has indeed recently been confirmed to contact DNA in ZitR_IL_ [21].

Few residues are involved in DNA major groove contacts in MarR proteins, and they are generally not conserved. A Thr residue conserved between ZitR_MG_ and OhrR sequences (S6 Fig) and structures (data not shown), Thr68_ZitR_, could be involved in such a contact. Indeed, in OhrR, its counterpart, Thr72_OhrR_ (α4), is known to interact with the DNA phosphate backbone of the major groove, *via* a main chain hydrogen interaction [29]. This Thr residue, which is also widely conserved among ZitR/AdcR sequences (Fig 1), has recently been confirmed to contact DNA major groove in the DNA-bound ZitR_IL_ [21], highly suggesting that it could have the same role in ZitR_MG_. In conclusion, structure superimposition to the available DNA-bound MarR proteins allowed us predicting ZitR_MG_ residues putatively involved in DNA binding, even though further experiments will be required to demonstrate their role.

The DNA-binding domain of ZitR_MG_ is connected to the Zn(II)-binding pocket by a hydrogen-bonding network, between α2 and α4 helices (Fig 2A), as is also the case in other MarR proteins [10].

#### The Zn(II)-binding pocket show subtle differences between ZitR and AdcR

**Fig 4:**
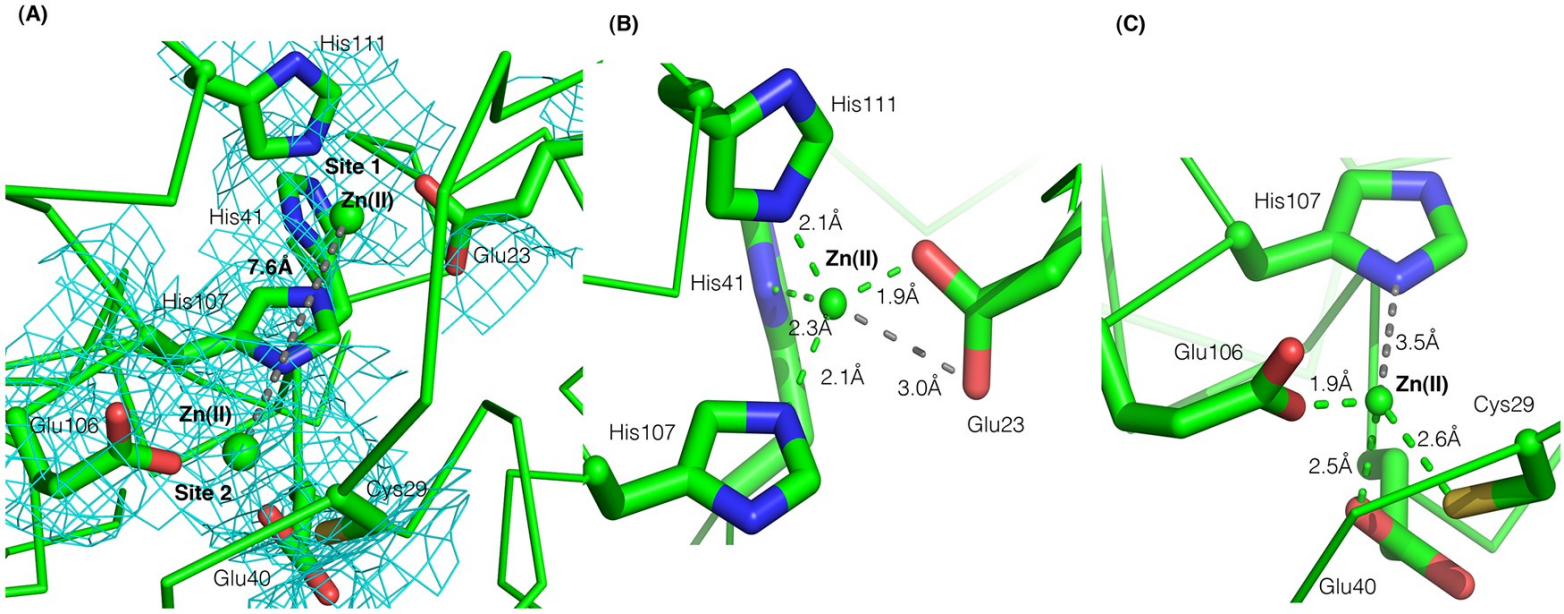
The Zn(II) metal binding pocket of holo-ZitR_MG_ dimer. Only the protomer A is shown in green. Zn(II) atoms are represented as small green spheres and their ligands as ball-and-sticks, with their N, O and S atoms respectively colored in blue, red and yellow. (A) Electron density map of the whole Zn(II) binding pocket. 2Fo-Fc contoured at 1.0σ is displayed as a cyan mesh. Internuclear Zn(II)-Zn(II) distance is indicated close to a grey dashed line between Zn(II) atoms. (B) Zn(II) binding site 1 and (C) Zn(II) binding site 2. Coordination bonds are represented as green dashed lines. A grey dashed line is shown between a residue atom and a Zn(II) atom when their distance is ≥ 3 Å.

The Zn(II)-binding pocket is made up by α2 and α5 helices, together with the extended α1-α2 loop of each protomer (Fig 1). Most residues involved in Zn(II) binding (see below) were buried or moderately exposed (S6 Fig), in marked contrast to residues proposed by structure alignment to be involved in DNA binding (see above). The two metal binding sites of ZitR_MG_ protein, like those of ZitR_IL_ [21] and AdcR_Spne_ [13], are located at opposite sides of the dimer and two Zn(II) ions are bound per protomer (Fig 2A, S4A and S4B Fig). At a first glance, the Zn(II)-binding sites of ZitR_MG_, ZitR_IL_ and AdcR_Spne_ proteins are highly similar, even though the internuclear Zn(II)-Zn(II) distance is shorter in ZitR_MG_ (7.3 Å -7.6 Å for protomers A and B; Fig 4A) than in ZitR_IL_ or AdcR_Spne_ (7.9 Å—8.1 Å for protomers A and B in both proteins; S7A and S7D Fig; only one Zn(II) ion per protomer could be determined in AdcR_GAS_ structure [20]). Zn(II)-coordinating residues are conserved and organized as globally similar binding sites between the two proteins (see below).

The Zn(II)-binding site 1 of ZitR_MG_ is composed of four residues conserved in ZitR/AdcR proteins (Fig 1). Glu23_ZitR_, His41_ZitR_ and His111_ZitR_ are moderately exposed, while His107_ZitR_ is buried (S6 Fig). Zn(II) is clearly coordinated by Oε1-Glu23_ZitR_, Nδ1-His41_ZitR_, Nε2-His107_ZitR_ and Nε2-His111_ZitR_, although a supplementary coordination involving a second atom of the first ligand (Oε2) has also been proposed in AdcR_Spne_ (see below) (Fig 4B; Table 3A). Our data are in good agreement with what has been observed for AdcR_Spne_ [13] and ZitR_IL_ [21], while in AdcR_GAS_ [20], only the last three ligands have been determined together with three water molecules (Table 3). In AdcR_Spne_, Zn(II) has been described as tetrahedrally coordinated by Oε1-Glu24_AdcR_, Nδ1-His42_AdcR_, Nε2-His108_AdcR_ and Nε2-His112_AdcR_ (S7B and S7E Fig; Table 3A) [13]. However, as previous X-ray absorption spectroscopy data had indicated that Zn(II) could be penta-coordinated in binding site 1 of AdcR_Spne_ [18], careful examination of 3D structure data revealed Oε2-Glu24_AdcR_ as a possible supplementary ligand. Indeed, Glu24_AdcR_ was proposed to be able, by a relatively small movement of its side chain, to achieve a bi-dentate coordination of Zn(II) *via* both Oε1 and Oε2, thus finally leading to Zn(II) penta-coordination [13]. Interestingly, the internuclear distances between the Zn(II) atom and each of its coordination residues are highly conserved among ZitR_MG_, ZitR_IL_ and AdcR_Spne_ proteins (Table 3A). Zn(II)-binding site 1 is therefore highly conserved between ZitR/AdcR proteins and contains at least four ligands (Oε1-Glu23, Nδ1-His41, Nε2-His107 and Nε2-His111 in ZitR_MG_), and possibly also a fifth one (Oε2-Glu23 in ZitR_MG_; Fig 4B, S7B and S7E Fig; Table 3A). Zn(II)-binding site 1 therefore displays the same geometry in all ZitR/AdcR proteins, even though its nature cannot yet be defined without ambiguity. Depending on whether Zn(II) is tetra- or penta-coordinated, Zn(II)-binding site 1 could respectively display a trigonal bipyramidal or a distorted tetrahedral geometry, as previously discussed in the case of AdcR_Spne_ [13]. Finally, our and previous structural data on ZitR_MG_ (this study), ZitR_IL_ [21] and AdcR_Spne_ [13, 18] are highly consistent.

**Table 3:** Comparison of Zn(II) coordination in ZnRR proteins.

**A** Zn(II)-binding site 1							
**ZitR**_**MG**_	**Å**	**ZitR**_**IL**_	**Å**	**AdcR**_**Spne**_	**Å**	**AdcR**_**GAS**_	**Å**
**Oε1-Glu23**	**1.9**	**Oε1-Glu24**	**2.3**	**Oε1-Glu24**	**2.0**	Oε1-Glu24 not determined	***Not Applicable***
Oε2-Glu23	3.0	Oε2-Glu24	2.9	Oε2-Glu24^a^	2.8	Oε2-Glu24 not determined	***Not Applicable***
**Nδ1-His41**	**2.3**	**Nδ1-His42**	**2.0**	**Nδ1-His42**	**2.0**	*Nδ1-His42*	*4*.*1*^b^
*Nε2-His41*^c^	*4*.*3*^b^	*Nε2-His42*^c^	*4*.*1* ^b^	*Nε2-His42*	*4*.*1* ^b^	**Nε2-His42**^c^	**2.4**
**Nε2-His107**	**2.1**	**Nε2-His108**	**2.0**	**Nε2-His108**	**2.1**	**Nε2-His108**	**2.3**
**Nε2-His111**	**2.1**	**Nε1-His112**	**2.1**	**Nε1-His112**	**2.0**	**Nε1-His112**	**2.3**
						3 H_2_O	**2.0, 2.2, 2.2**
**B** Zn(II)-binding site 2							
**ZitR**_**MG**_	**Å**	**ZitR**_**IL**_	**Å**	**AdcR**_**Spne**_	**Å**	**AdcR**_**GAS**_	**Å**
**S-Cys29**	**2.6**	**S-Cys30**	**2.5**	**S-Cys30**	**2.3**	S-Cys30	***Not Applicable*** ***No Zn(II)***
**Oε1-Glu40**	**2.5**	**Oε1-Glu41**	**2.2**	**Oε1-Glu41**	**2.0**	Oε1-Glu41	***Not Applicable*** ***No Zn(II)***
**Oε1-Glu106**	**1.9**	**Oε1-Glu107**	**2.0**	**Oε1-Glu107**	**2.0**	Oε1-Glu107	***Not Applicable*** ***No Zn(II)***
		H_2_O	1.8	H_2_O	2.1		

The distance between a zinc atom and each of its ligands is indicated for each site (site 1 in A and site 2 in B) of the following ZnRR proteins of the MarR superfamily: ZitR_MG_ (this study, 6FI9), ZitR_IL_ (5YHX), AdcR_Spne_ (3TGN) and AdcR_GAS_ (5JLS).

^a^Oε2-Glu24 has been proposed in AdcR_Spne_ to be a putative fifth ligand for Zn(II) in the binding site 1 [13] according to X-ray absorption spectroscopy data [18].

^b^Ligands whose distance to Zn(II) atom is > 3 Å are indicated in italic.

^c^Nε2-His42 is the Zn(II) ligand in ZitR_IL_ site 1 only when site 2 is not occupied, and switches to Nδ1-His42 upon binding of a second Zn(II) to site 2 [21]. Nε2-His42 in AdcR_GAS_ [20] is closer to Zn(II) bound in site 1 than Nδ1-His42.

Overall, the Zn(II) binding site 2 is also well conserved among ZitR/AdcR proteins. The three Zn(II) ligands in ZitR_MG_ site 2: SH-Cys29_ZitR_, Oε1-Glu40_ZitR_ (a buried residue, S6 Fig), and Oε1-Glu106_ZitR_ (Figs 1 and 4C; Table 3B), are the same as in ZitR_IL_ and AdcR_Spne_ (S7C and S7F Fig; Table 3B). The unique Cys residue of ZitR/AdcR proteins (SH-Cys29 in ZitR_MG_) is therefore confirmed to be a Zn(II) ligand in ZitR_MG_ (this study) like in ZitR_IL_ [13, 21], and AdcR_Spne_ [13], in contrast to initial findings for the latter protein [18]. Yet, a water molecule, which is used as a fourth Zn(II) ligand in AdcR_Spne_ [13] and ZitR_IL_ [21], has not been observed here in ZitR_MG_. It cannot be excluded (Table 3B) that such a water molecule could be part of Zn(II)-binding site 2 in ZitR_MG_ under other conditions. Alternatively, as the distance between the Zn(II) atom of site 2 and one His residue of site 1 is slightly smaller in ZitR_MG_ (3.5 Å for Zn(II)- His107_ZitR_) (this study) than in ZitR_IL_ (4.0 Å for Zn(II)-His108_ZitR_) [21] or AdcR_Spne_ (4.1 Å for Zn(II)-His108_AdcR_) [13]) (Table 3B), the whole Zn(II)-binding pocket could display subtle folding differences among ZitR/AdcR proteins.

The mechanism of Zn(II)-binding and its effect on DNA-binding have recently been clarified in ZitR_IL_. In this protein, the binding of a second Zn(II) atom at site 2 involved a switch in Zn(II) coordination at the high affinity site 1, between the two nitrogen atoms of His42, leading to the reorganization of the wHTH domain into an optimal DNA-binding conformation [21]. The binding cooperativity between the two Zn(II) binding sites enables ZitR_IL_ to adapt to a broad range of zinc fluctuation that results in a fine-tuned control of transcriptional regulation. In other MarR proteins, like AdcR_Spne_, the two Zn(II) sites have also been found to display different affinities, and the high and low affinity sites were respectively proposed to be a regulatory site and a site of unknown function [13, 18]. In ZitR_MG_ (this study), Zn(II) binding site 2 could serve to modulate DNA-binding activity, as proposed for AdcR_Spne_ [13]. Alternatively, as our previous *in vivo* data indicate that ZitR_MG_ functions as a repressor in a wide concentration range, from repletion to toxicity [16], Zn(II) binding at site 2 could allow modulating gene expression in response to high to toxic Zn(II) concentrations, in ZitR_MG_ like in ZitR_IL_ [21]. Further studies would be needed to definitely establish the function of Zn(II) binding in ZitR_MG_ protein.

Intriguingly, similar properties have also been described for Zur metallo-regulators belonging to the Fur superfamily and used by most bacterial species. In *Streptomyces coelicolor* Zur protein, there are two regulatory sites (M and D) that regulate DNA-binding in different ways, by an on-off switch and a fine-tuner respectively [36]. In *B*. *subtilis* Zur protein, there is a sequential negative binding cooperativity between the regulatory Zn(II) binding sites, possibly allowing this protein to sense a broader range of intracellular Zn(II) concentrations [37]. Understanding why gene regulation by Zn(II) is performed by ZitR/AdcR proteins in the *Streptococaceae* group and Zur proteins in almost all other bacterial species will deserve further investigations.

## Conclusions

In this study, we have characterized ZitR_MG_, a metallo-regulator of the MarR superfamily. In solution, ZitR_MG_ has been shown to be a stable dimer able to bind, as expected [16], both Zn(II) and double-stranded DNA fragments bearing the palindromic **TTAAC**YR**GTTAA** recognition sequence [16, 19]. We have determined the structure of holo-ZitR_MG_ by X-ray crystallography. ZitR_MG_ is the fourth ZnRR in MarR superfamily whose structure has been determined. The overall 3D fold of ZitR_MG_ best matches that of ZitR_IL_ [21], is highly similar to that of AdcR_Spne_ [13] and, to a lesser extent, to that of AdcR_GAS_ [20], and is related to that of other MarR members.

Interestingly, when holo-ZitR_MG_ and a few Zn(II)-independent regulators of the MarR superfamily have been compared, the former has been found to be closer to DNA-bound state of the MarR members than to its DNA-free state. This suggests that Zn(II) binding could drive modifications of the quaternary structure to efficiently accommodate DNA in ZitR_MG_, as previously shown in *E*.*coli* MarR proteins [9] and recently in ZitR_IL_ [21].

## Supporting information

S1 Table ZitR_MG_ DNA-binding as revealed by ITC experiments. (PDF)

S1 Fig Purified ZitR_MG_ is a dimer. SECMALS analysis of untreated (in black) and EDTA-treated ZitR_MG_ protein (in red) using a KW-803 column. Elution profiles are represented as a function of the molar mass. The molar mass and the hydrodynamic radius of EDTA-treated ZitR_MG_ have been calculated from light scattering and refractometry data and found to be 32.1 ± 0.3 kDa and 2.3 ± 0.1 nm, respectively.
(TIF)

S2 Fig Purified ZitR_MG_ is a stable protein. ZitR_MG_ has been characterized by circular dichroism (CD): Ellipticity (θ) in millidegrees (mdeg) is shown as a function of the wavelength (from 190 to 270 nm). CD temperature scans by steps of 10 °C, from 20 °C to 80 °C are shown (the color of the scan is different as a function of the temperature, and the correspondence is shown on the right). In the insert, ellipticity (θ) in millidegrees (mdeg) is shown as a function of temperature at either 208 nm (full square) or 222 nm (empty square). The inflexion point of both curves is the ZitR_MG_ melting temperature (Tm), and it is of around 75 °C.
(TIF)

S3 Fig Purified ZitR_MG_ is able to bind palindromic double-stranded DNA fragments. ITC measurements of ZitR_MG_ interaction with two different palindromic dsDNA fragments. (A) Palindrome 1 overlapping the -35 box of *Pzit* promotor and (B) Palindrome 2 overlapping the -10 box of *Pzit* promotor (see S1 Table for the sequences of forward oligonucleotides). The top panels show the raw data, the heat (microcalories.second^-1^) generated by each injection of DNA during titration experiments as a function of time (minutes). The bottom panels show the binding isotherms created by plotting the heat as a function of protein concentration and the fitting to theoretical curves. A representative experiment is shown in each case. These figures have been created with Origin (OriginLab, Northampton, MA).
(TIF)

S4 Fig Structure conservation between ZnRR holo-dimers in the MarR superfamily. The structure of ZitR_MG_ holo-dimer (this study, 6FI9, in green) and that of either (A) ZitR_IL_ (5HYX, in magenta) or (B) AdcR_Spne_ (3TGN, in blue) holo-dimers have been superimposed. Each protein is shown in ribbon representation with protomer A and B in dark and light color respectively, and labelled N and C-terminus. Zn(II) atoms are shown as spheres of the same color as the protein.
(TIF)

S5 Fig Structure conservation between ZitR proteins in the wHTH DNA-binding domain. The structure of holo-ZitR_MG_ (this study, 6FI9, in green) and that of either (A) WT DNA-bound ZitR_IL_ (5YI2, in magenta) or (B) C30S DNA-bound ZitR_IL_ (5YI3, in violet) holo-forms have been superimposed. Only a zoom on a small region of the wHTH DNA-binding domain of each protein (protomer A) is shown in ribbon representation. In the last two cases, DNA is in light color. Residues known to contact DNA in ZitR_IL_ and the residues aligned to them in ZitR_MG_ are represented as ball-and-sticks, in dark or light color depending on whether they are accessible or moderately accessible, with their N and O atoms colored in blue and red respectively, and labelled.
(TIF)

S6 Fig Sequence alignment between ZitR_MG_ protein and other MarR superfamily members. The sequence of ZitR_MG_ (this study, 6FI9), and that of other MarR members (non-ZnRR proteins): RovA (4AIJ), SlyA (3Q5F), and OhrR (1Z9C) have been multi-aligned like in Fig 1. Secondary elements of ZitR_MG_ protein are displayed above its sequence, and the accessibility of its residues is shown below the alignment using the following color code: dark blue, cyan and white respectively indicate fully accessible, moderately accessible and buried residues. Residues identical in all proteins are shown in white characters in a red background. Residues conserved in at least one, and up to three of the other MarR proteins are indicated as following: ‘3’, ‘2’ and ‘1’ characters under the alignment respectively label residues conserved in all the three non-ZnRR MarR proteins (RovA, SlyA and OhR), in two of them (RovA and SlyA), or in only one of them OhrR.
(TIF)

S7 Fig Comparison of the Zn(II) binding sites between ZnRR proteins. Zn(II) binding domains are compared between ZitR_MG_ holo-dimer (in green) and either ZitR_IL_ (in magenta, A-C) or AdcR_Spne_ (in blue, D-F) holo-dimers (only protomer A is shown of each protein). The whole metal binding pocket (A, D), Zn(II) binding site 1 (B, E), and 2 (C, F) are represented. Zn(II) atoms are shown as spheres of the same color as the protein. Water molecules are represented as red spheres. Residues involved in Zn(II) binding are in ball-and-sticks with N, O and S atoms respectively colored in blue, red and yellow. Zn(II)-Zn(II) inter-nuclear distance is indicated close to a grey dashed line (A, D). In each comparison, only the residues of ZitR_IL_ or AdcR_Spne_ proteins are labelled.
(TIF)
