# Supplementary material for: Biophysical and structural characterization of a zinc-responsive repressor of the MarR superfamily
Source: PLoS One. 2019 Feb 12;14(2):e0210123. doi: 10.1371/journal.pone.0210123 (PMC6372160; doi:10.1371/journal.pone.0210123)
Supplement: S1 Table — (PDF) [file pone.0210123.s001.pdf]

**dsDNA fragment**	**ZitR$_{MG}$ dimer: DNA stoichiometry (n)**	**Binding affinity K_d (M.L^1)**	**Enthalpy changes ΔH (kcal.mol^{-1})**
**Palindrome 1 of Pzit promoter -35 box** AATG**TTAAC**TG***G****TT**GACA*T[a]	1.2[b]	0.2.10^{-6}[b]	11.3[b]
**Palindrome 2 of Pzit promoter -10 box** *A**TA**A**T***TAA**C**CA**GT**AAA**C**T**A**A[a]	1.5[b]	0.6.10^{-6}[b]	10.6[b]

[a]***Each oligonucleotide is an imperfect palindrome: complementary bases of the inverted repeat are in bold [15, 18]. The -35 and -10 boxes of Pzit promoter, which partially overlap each palindromic sequence, are in italics (the 5' 'T' base of the -10 box is missing in Palindrome 2) [15].***
[b]***The average values between two independent measurements are shown.***
