# Supplementary figures and images for: Biophysical and structural characterization of a zinc-responsive repressor of the MarR superfamily
Source: PLoS One. 2019 Feb 12;14(2):e0210123. doi: 10.1371/journal.pone.0210123 (PMC6372160; doi:10.1371/journal.pone.0210123)

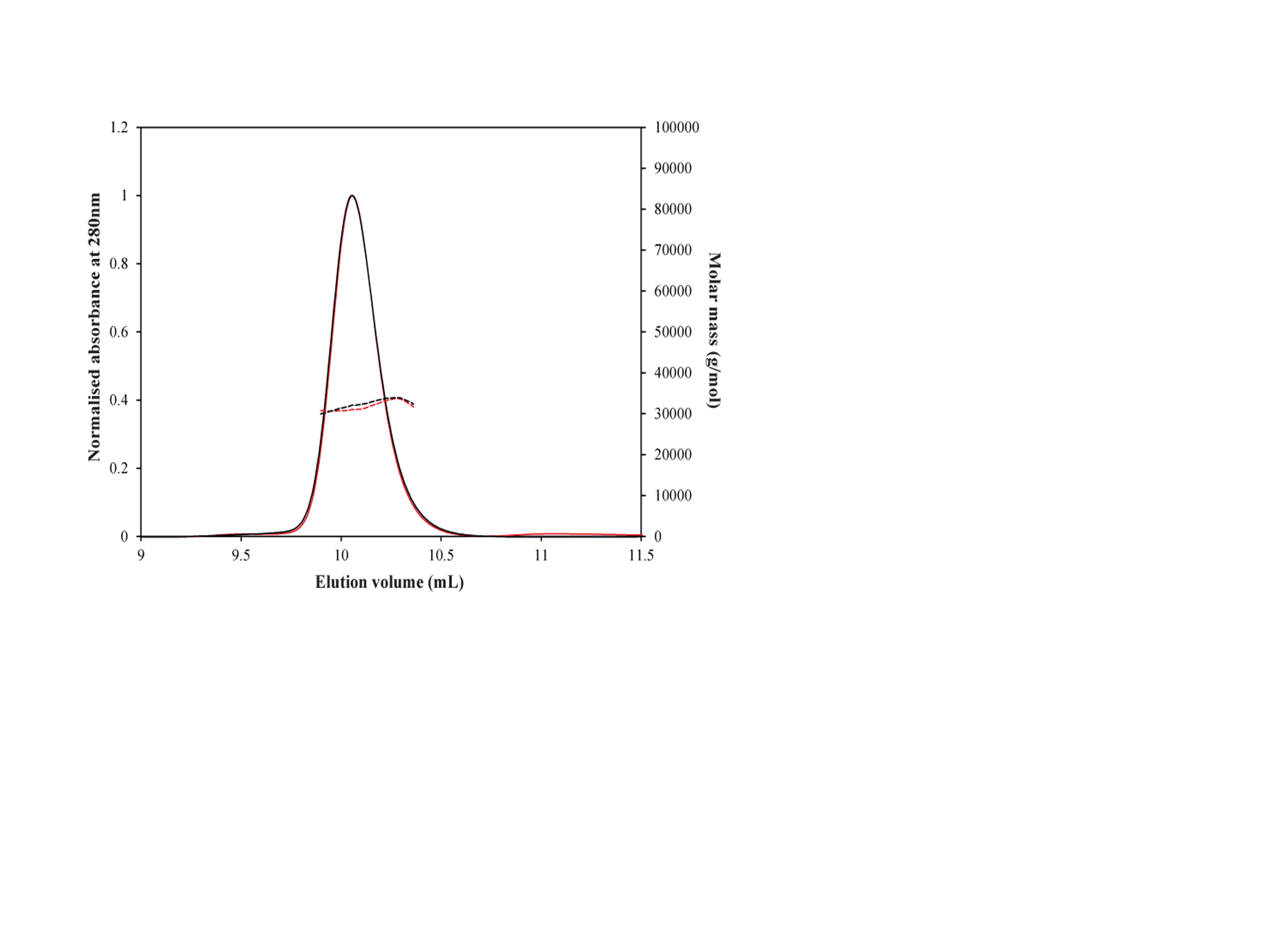

Supplement: S1 Fig — SECMALS analysis of untreated (in black) and EDTA-treated ZitRMG protein (in red) using a KW-803 column. Elution profiles are represented as a function of the molar mass. The molar mass and the hydrodynamic radius of EDTA-treated ZitRMG have been calculated from light scattering and refractometry data and found to be 32.1 ± 0.3 kDa and 2.3 ± 0.1 nm, respectively. (TIF) [file pone.0210123.s002.tif]

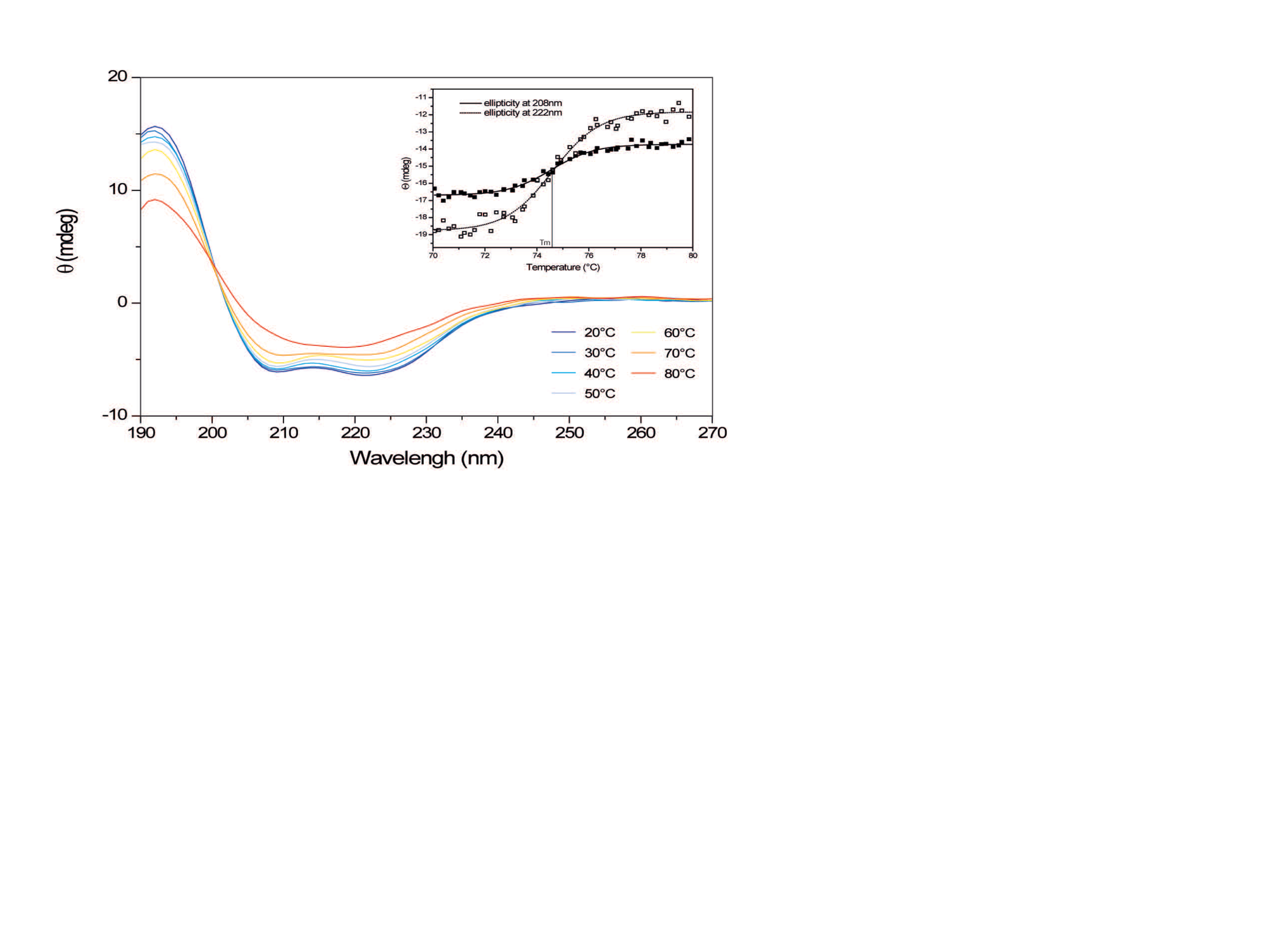

Supplement: S2 Fig — ZitRMG has been characterized by circular dichroism (CD): Ellipticity (θ) in millidegrees (mdeg) is shown as a function of the wavelength (from 190 to 270 nm). CD temperature scans by steps of 10 °C, from 20 °C to 80 °C are shown (the color of the scan is different as a function of the temperature, and the correspondence is shown on the right). In the insert, ellipticity (θ) in millidegrees (mdeg) is shown as a function of temperature at either 208 nm (full square) or 222 nm (empty square). The inflexion point of both curves is the ZitRMG melting temperature (Tm), and it is of around 75 °C. (TIF) [file pone.0210123.s003.tif]

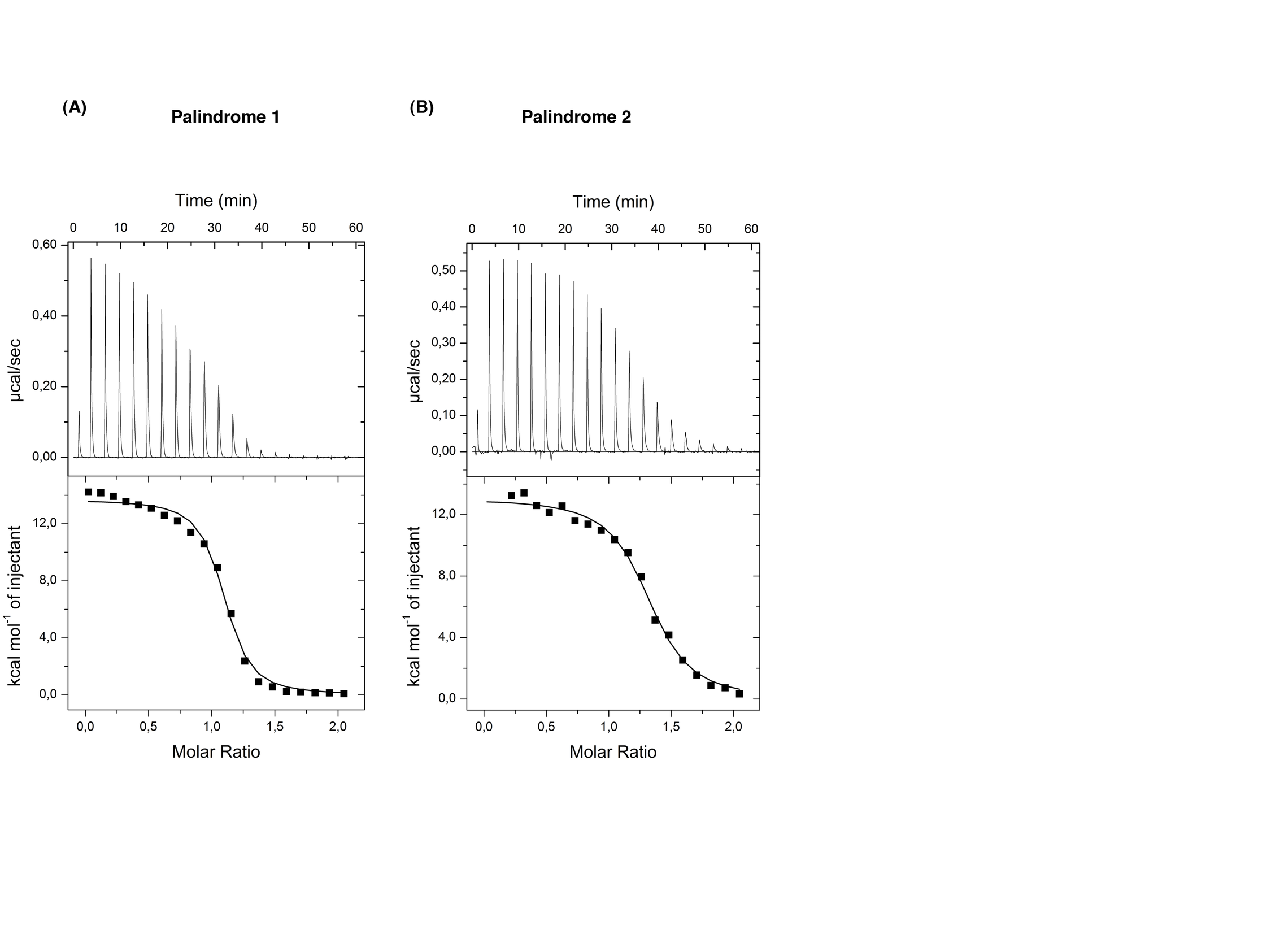

Supplement: S3 Fig — ITC measurements of ZitRMG interaction with two different palindromic dsDNA fragments. (A) Palindrome 1 overlapping the -35 box of Pzit promotor and (B) Palindrome 2 overlapping the -10 box of Pzit promotor (see S1 Table for the sequences of forward oligonucleotides). The top panels show the raw data, the heat (microcalories.second-1) generated by each injection of DNA during titration experiments as a function of time (minutes). The bottom panels show the binding isotherms created by plotting the heat as a function of protein concentration and the fitting to theoretical curves. A representative experiment is shown in each case. These figures have been created with Origin (OriginLab, Northampton, MA). (TIF) [file pone.0210123.s004.tif]

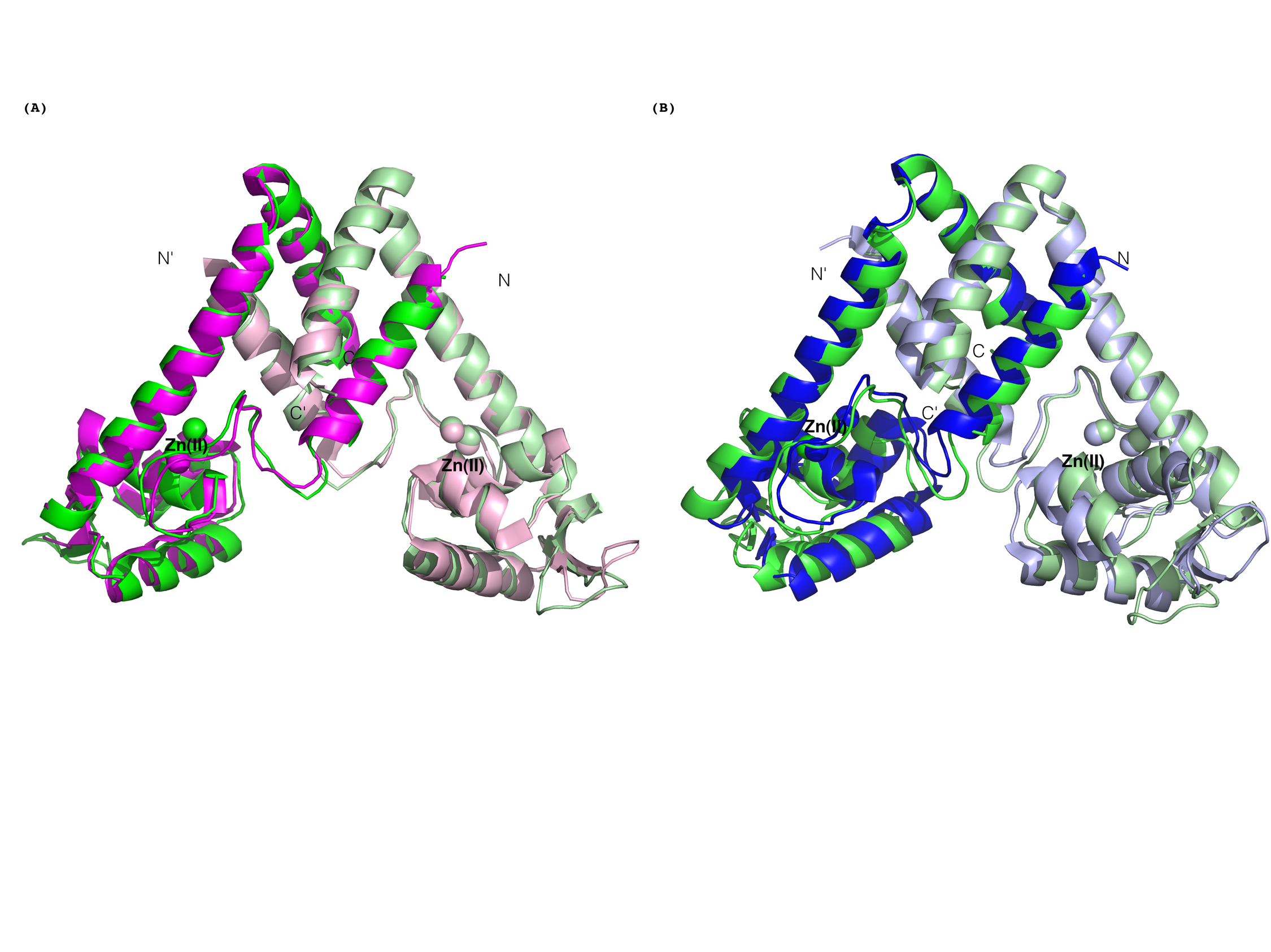

Supplement: S4 Fig — The structure of ZitRMG holo-dimer (this study, 6FI9, in green) and that of either (A) ZitRIL (5HYX, in magenta) or (B) AdcRSpne (3TGN, in blue) holo-dimers have been superimposed. Each protein is shown in ribbon representation with protomer A and B in dark and light color respectively, and labelled N and C-terminus. Zn(II) atoms are shown as spheres of the same color as the protein. (TIF) [file pone.0210123.s005.tif]

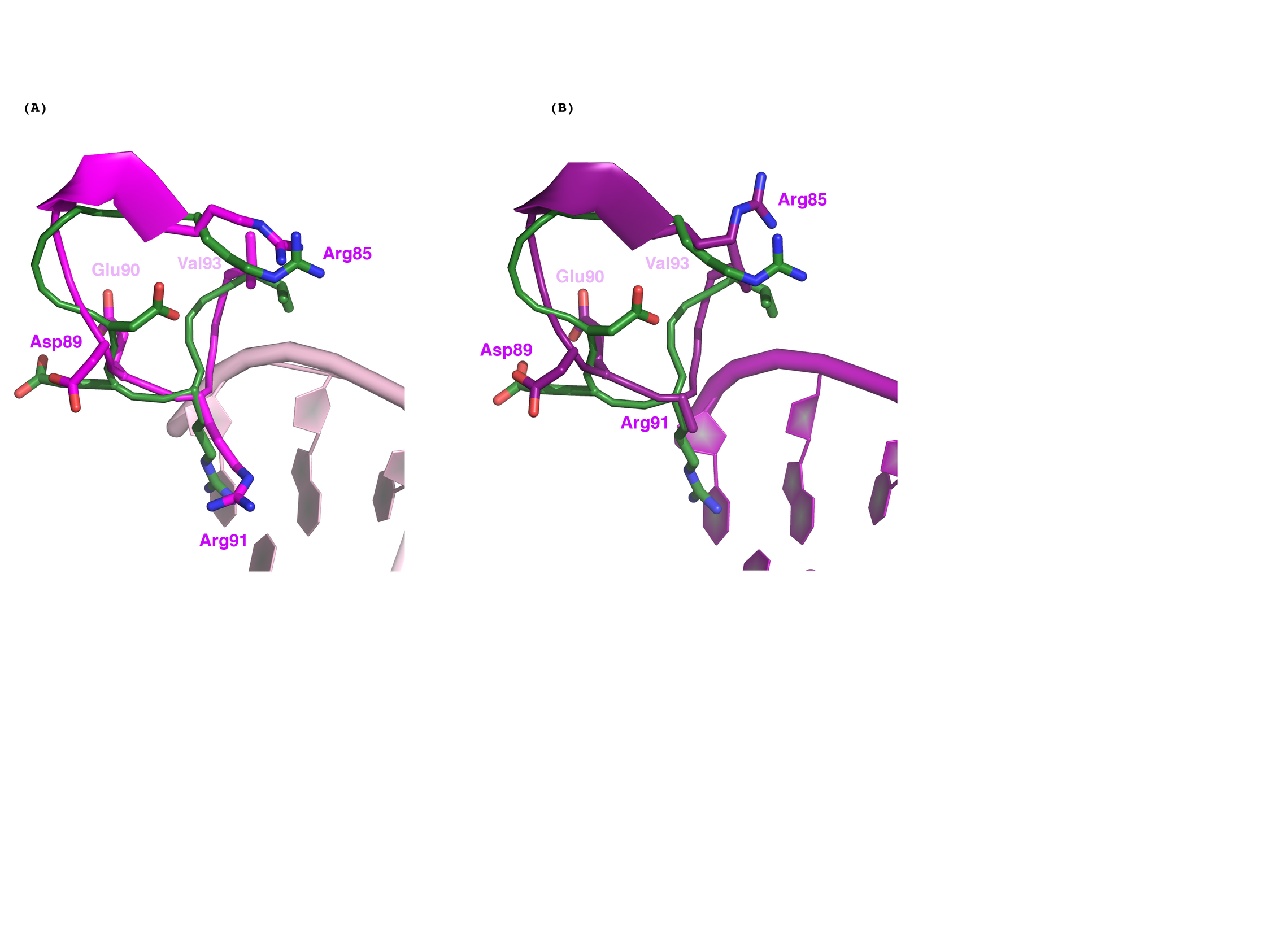

Supplement: S5 Fig — The structure of holo-ZitRMG (this study, 6FI9, in green) and that of either (A) WT DNA-bound ZitRIL (5YI2, in magenta) or (B) C30S DNA-bound ZitRIL (5YI3, in violet) holo-forms have been superimposed. Only a zoom on a small region of the wHTH DNA-binding domain of each protein (protomer A) is shown in ribbon representation. In the last two cases, DNA is in light color. Residues known to contact DNA in ZitRIL and the residues aligned to them in ZitRMG are represented as ball-and-sticks, in dark or light color depending on whether they are accessible or moderately accessible, with their N and O atoms colored in blue and red respectively, and labelled. (TIF) [file pone.0210123.s006.tif]

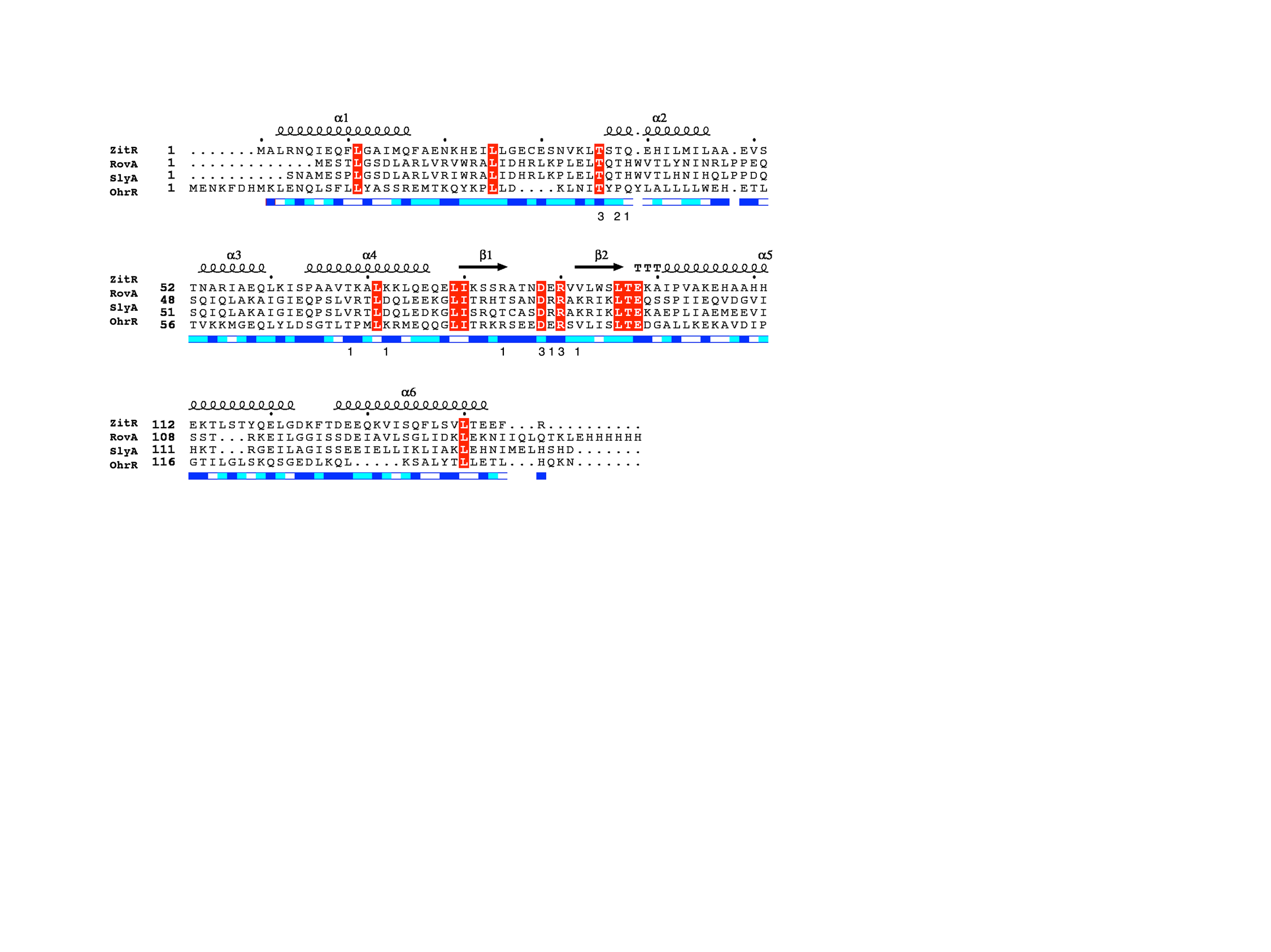

Supplement: S6 Fig — The sequence of ZitRMG (this study, 6FI9), and that of other MarR members (non-ZnRR proteins): RovA (4AIJ), SlyA (3Q5F), and OhrR (1Z9C) have been multi-aligned like in Fig 1. Secondary elements of ZitRMG protein are displayed above its sequence, and the accessibility of its residues is shown below the alignment using the following color code: dark blue, cyan and white respectively indicate fully accessible, moderately accessible and buried residues. Residues identical in all proteins are shown in white characters in a red background. Residues conserved in at least one, and up to three of the other MarR proteins are indicated as following: ‘3’, ‘2’ and ‘1’ characters under the alignment respectively label residues conserved in all the three non-ZnRR MarR proteins (RovA, SlyA and OhR), in two of them (RovA and SlyA), or in only one of them OhrR. (TIF) [file pone.0210123.s007.tif]

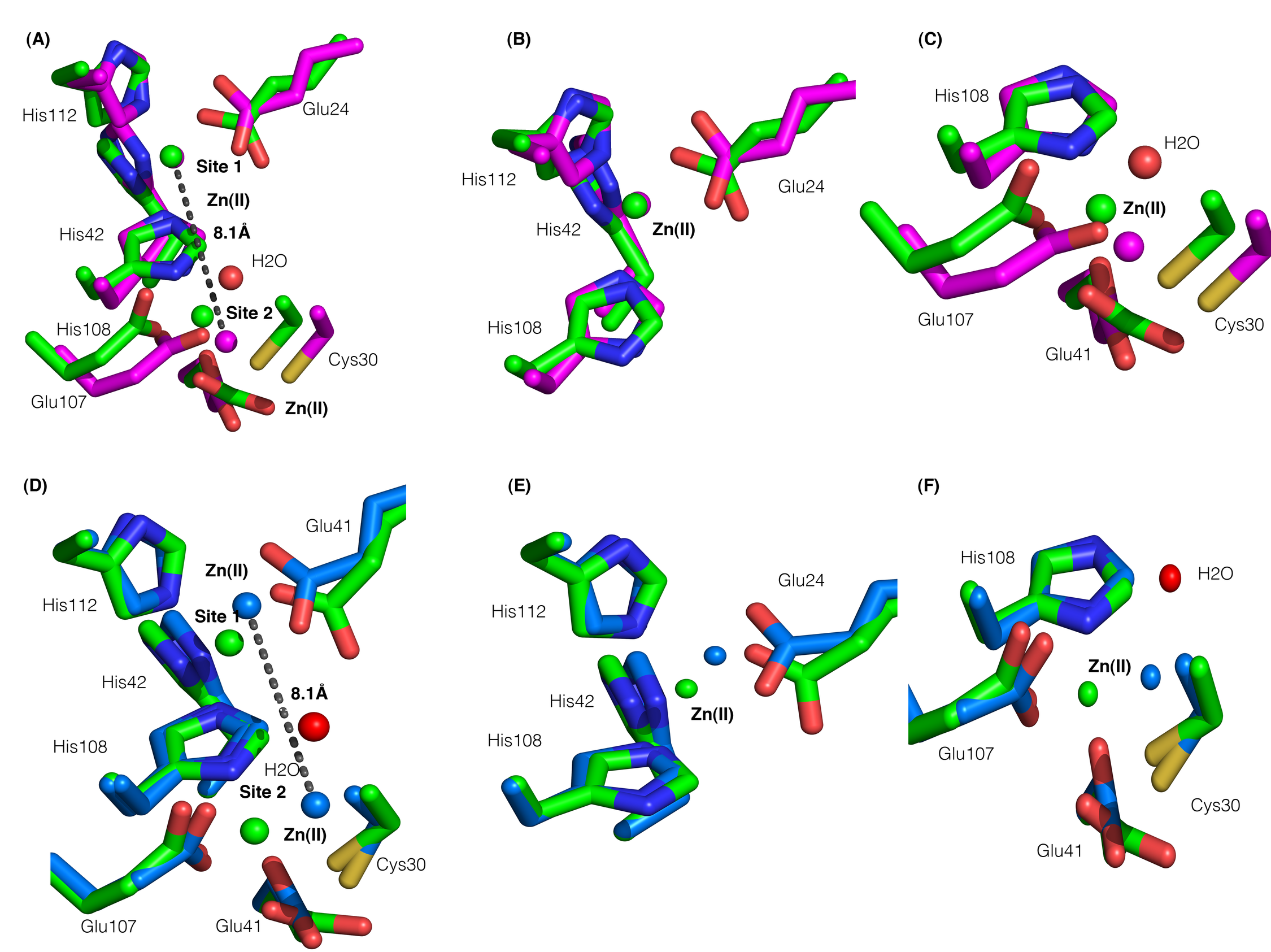

Supplement: S7 Fig — Zn(II) binding domains are compared between ZitRMG holo-dimer (in green) and either ZitRIL (in magenta, A-C) or AdcRSpne (in blue, D-F) holo-dimers (only protomer A is shown of each protein). The whole metal binding pocket (A, D), Zn(II) binding site 1 (B, E), and 2 (C, F) are represented. Zn(II) atoms are shown as spheres of the same color as the protein. Water molecules are represented as red spheres. Residues involved in Zn(II) binding are in ball-and-sticks with N, O and S atoms respectively colored in blue, red and yellow. Zn(II)-Zn(II) inter-nuclear distance is indicated close to a grey dashed line (A, D). In each comparison, only the residues of ZitRIL or AdcRSpne proteins are labelled. (TIF) [file pone.0210123.s008.tif]
